# The Effect of the COVID-19 Pandemic on Physicians’ Use and Perception of Telehealth: The Case of Lebanon

**DOI:** 10.3390/ijerph17134866

**Published:** 2020-07-06

**Authors:** Samar Helou, Elie El Helou, Victoria Abou-Khalil, Jad Wakim, Jeanine El Helou, Alain Daher, Charline El Hachem

**Affiliations:** 1 Global Center for Medical Engineering and Informatics, Osaka University, Osaka 565-0871, Japan; 2 Faculty of Medicine, Saint Joseph University, Beirut 1107 2180, Lebanon; ehelou@gmail.com (E.E.H.); wakim1@hotmail.com (J.W.); jeanine.helouelsaliba@usj.edu.lb (J.E.H.); daheralain@hotmail.com (A.D.); charlineeh@gmail.com (C.E.H.); 3 Academic Center for Computing and Media Studies, Kyoto University, Kyoto 606-8315, Japan; v.aboukhalil@gmail.com

**Keywords:** telehealth, telemedicine, perceptions, use, physicians, Lebanon, COVID-19, pandemic, change

## Abstract

The COVID-19 pandemic forced physicians to quickly adapt and find ways to provide their usual offline services by using online tools. We aimed to understand how physicians adapted to the sudden need for telehealth and if their perception of telehealth changed due to their experience during the COVID-19 pandemic. We conducted an exploratory sequential mixed-methods study. We interviewed five Lebanese physicians and thematically analyzed the interviews. We developed a questionnaire based on the analysis results and administered it online to physicians in Lebanon. In total, 140 responses were collected. We found that, during the COVID-19 pandemic, physicians engaged in more telehealth activities in the realms of telemedicine, public awareness, continuing medical education, research, administration, and teaching. They also expanded their repertoire of information-technology tools. Our results also show that there was a significant shift in the physicians’ perceptions, indicating greater openness and willingness to adopt telehealth services. However, a significant amount of skepticism and uncertainty regarding telemedicine remains, especially concerning its efficiency, safety, and the adequacy of existing regulations. Based on our findings, we offer recommendations for health IT policy makers, developers, and researchers, to sustain the continuity of telehealth activities beyond the COVID-19 pandemic.

## 1. Introduction

On 21 February 2020, Lebanon confirmed its first case of Corona Virus Disease 2019 (COVID-19) [1]. On 15 March 2020, Lebanon initiated a nationwide lockdown, aiming to contain the spread of the novel coronavirus [2]. Soon after, the Lebanese Order of Physicians encouraged physicians to provide healthcare services remotely, when deemed possible [3]. This request was one of the measures taken to increase social distancing and home confinement. 

Prior to the COVID-19 pandemic, the use of telehealth in Lebanon was limited [4], even though Lebanon has high levels of smartphone (80%) and internet (76%) penetration [5]. Telehealth activities included small-scale interventions targeted at refugee and underserved populations [6], as well as the use of telemedicine in well-defined medical tasks [7,8]. However, telehealth services can provide benefits to all the residents of Lebanon in terms of increasing access to healthcare services, raising public health awareness, and providing healthcare training. 

Indeed, Lebanese and foreigners residing in Lebanon have multiple barriers that can hinder their access to healthcare. These barriers can be geographical, cultural, societal, organizational, economical, and sometimes political. In terms of geographical barriers, healthcare services are mainly centralized within and around the country’s capital city, Beirut [9]. Cultural and societal barriers include limited awareness and health literacy, as well as widespread social and religious stigmas that reduce the offer of, and demand for, certain healthcare services, such as mental, sexual, and reproductive health services in certain communities. This is heightened by the coexistence of multiple religious sects and ethnic groups [10]. In terms of economic barriers, prohibitive services costs and the lack of universal health coverage can prevent patients from receiving needed care [11]. Finally, individuals with lower political involvement may have less access to healthcare services [12], a phenomena that can be attributed to the sectarian structures and a widespread culture of political clientelism. In this scheme, the adoption of telehealth can contribute to democratizing access to healthcare, increasing awareness, improving education, and lowering the costs of healthcare. In addition, the wide adoption and use of telehealth services can alleviate other social problems, such as traffic and pollution [13]. This is highly relevant in the Lebanese context [14], where traffic and air pollution from vehicles are, and will probably remain, unresolved problems due to a lack of top-down solutions.

Although telehealth can provide many benefits, the adoption of telehealth services remains limited in the Lebanese context. Previous research highlighted the need to understand the physicians’ perceptions and attitudes toward telehealth [15,16,17] and noted that the limited adoption of telehealth is mainly attributed to physicians’ unwillingness to use telehealth services [18]. In the case of Lebanon, not much is known about physicians’ needs and perceptions of telehealth. Understanding the ways in which they engage in telehealth can highlight their needs and inform the design of tools to fulfill those needs. Moreover, it is necessary to understand the physicians’ perceptions, in order to ensure their active participation in future telehealth initiatives, and consequently increase the chances of success of these initiatives.

The sudden need to use telehealth during the COVID-19 pandemic forced physicians to temporarily conduct their usual offline activities by using online tools. This offers an opportunity to understand the physicians’ telehealth needs by looking at how they adapted their offline activities. Moreover, physicians in Lebanon gained new experiences in telehealth which may have affected their perceptions of it. The aim of this study is to answer the following research questions.

- RQ1: How did Lebanese physicians adjust their use of telehealth during the COVID-19 pandemic?
- RQ2: How did the experience of using telehealth during the COVID-19 pandemic affect the perceptions of Lebanese physicians regarding telehealth?

By answering these research questions, we can understand the telehealth needs of physicians in Lebanon and their new perceptions regarding it. Based on this understanding, we can design solutions and policies that address the barriers and exploit the facilitators for telehealth from the physicians’ perspective. 

## 2. Materials and Methods 

**Figure 1:**
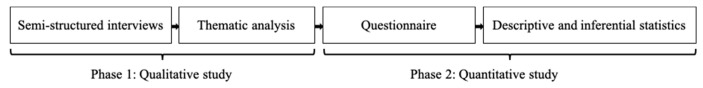
The methodology used in this study.

We used an exploratory sequential mixed methods approach—a qualitative study, followed by a quantitative study. The results of the initial qualitative study informed the design of the quantitative study. The study was approved by the ethics committee of Saint Joseph University, Lebanon. The methodology is shown in Figure 1.

### 2.1. Qualitative Study

First, we conducted semi-structured interviews with five Lebanese physicians. In the interviews, we asked the participants about their use and perceptions of telehealth prior to, and during, the COVID-19 pandemic. A guide for the semi-structured interview is shown in Appendix A. 

The interviews were transcribed and thematically analyzed, using QDA miner, a qualitative analysis software. The analysis was conducted by following the six-phase guide of Braun and Clarke [19]. The analysis resulted in a list of themes describing the types of telehealth the physicians engaged in, the tools they used for telehealth, and their perceptions regarding telehealth. 

### 2.2. Quantitative Study

The questionnaire was designed based on the results of the thematic analysis where each subtheme was mapped to a question. The questionnaire contained questions about the following:

- The age, gender, and specialty of the physicians;
- The types of telehealth activities they engaged in before the COVID-19 pandemic, and the activities they engaged in more during the COVID-19 pandemic;
- The tools they used for telehealth before the COVID-19 pandemic, and the tools they used more during the COVID-19 pandemic;
- Their perceptions regarding various aspects of telehealth before, and during, the COVID-19 pandemic.

The questionnaire was administered online as a Google Form in English and took approximately ten minutes to complete. The target study population for the questionnaire was physicians practicing in Lebanon. The respondents were recruited by using a snowball sampling approach initiated by the authors of the paper. Snowball sampling allowed us to directly access Lebanese physicians by way of interpersonal relationships and connections and to quickly gather a large number of responses. The responses were collected over a period of three days, from 14 May to 16 May 2020.

To understand the use of telehealth before and during the COVID-19 pandemic, we analyzed the collected data by using descriptive statistics. To understand if the physicians changed their perceptions regarding telehealth, we analyzed the data by using non-parametric inferential statistics, namely Mann–Whitney U tests.

## 3. Results

### 3.1. Themes Identified in the Interviews

Based on the thematic analysis of the interviews, we found that the telehealth activities of physicians in Lebanon fall into six major themes: telemedicine, public awareness, continuing medical education, research, administration, and teaching. Regarding the perceptions of telehealth, our analysis resulted in nine themes: need for telehealth, health-related discussions on social media, effectiveness of telemedicine, efficiency of telemedicine, patient satisfaction with telemedicine, safety of telemedicine, medicolegal aspects, renumeration for telemedicine services, and management of telemedicine practice. The themes, subthemes, and supporting quotes from the interviews are shown in Table 1.

### 3.2. Questionnaire Respondents

In total, 140 physicians based in Lebanon responded to the questionnaire. The respondents’ characteristics are shown in Table 2.

### 3.3. Telehealth Activities before the COVID-19 Pandemic

**Figure 2:**
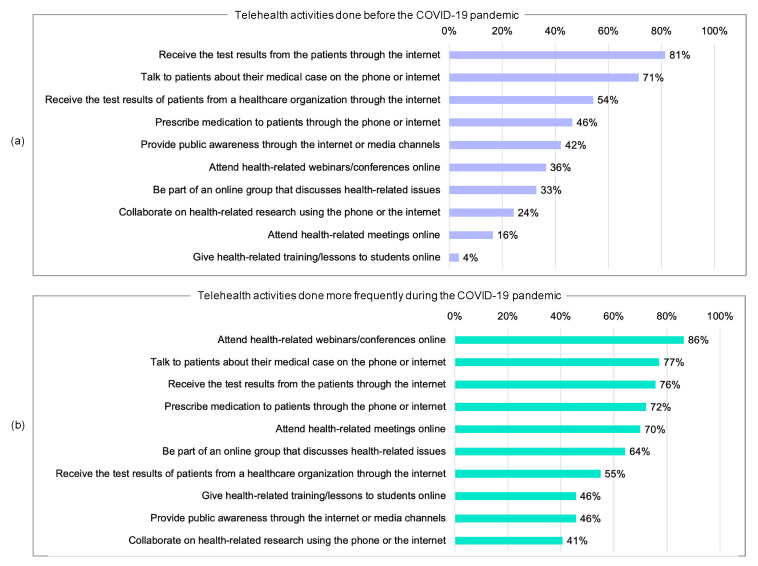
(**a**) Telehealth activities done before the COVID-19 pandemic; (**b**) telehealth activities done more frequently during the COVID-19 pandemic.

Figure 2a shows the types of telehealth activities that the physicians performed before the COVID-19 pandemic. The numbers indicate the proportions of physicians that engaged in those activities. 

In terms of telemedicine, the results show that, prior to the COVID-19 pandemic, the majority of participants received test results from the patients over the internet (81%), talked to patients about their medical cases over the phone or the internet (71%), and received test results from test centers over the internet (54%). Around half of the physicians prescribed medications to their patients over the phone or internet (46%). 

In terms of public awareness, we found that a large number provided public medical awareness through the internet or other media channels, such as TV and radio appearances (42%). 

In terms of continuous medical education, around a third of the participants attended health-related webinars or conferences online (36%) or were part of an online group that discusses health-related issues (33%).

In terms of research, administrative, and teaching activities, almost a quarter did research collaborations by using the phone or the internet (24%), and less than a fifth of physicians attended health-related administrative meetings online (16%). A small minority gave health-related training and lessons to students online (4%).

### 3.4. Telehealth Activities That Were Done More Frequently during the COVID-19 Pandemic

Figure 2b shows the types of telehealth activities that were performed more frequently during the COVID-19 pandemic. The numbers indicate the proportion of physicians who increased their engagement in those activities. The results show that, in general, physicians increased their telehealth activities during the COVID-19 pandemic. In terms of telemedicine, the majority of physicians talked more frequently to their patients on the phone or using internet apps (77%), received more tests from their patients (76%) and from test centers (55%) over the internet, and prescribed medications to their patients electronically (72%).

In terms of continuing medical education, the majority of the participants attended more health-related webinars or conferences online (86%) or increased participation in online groups that discuss health-related issues (64%). 

In terms of raising public awareness, around half of the participants provided more public medical awareness through the internet or other media channels, such as TV and radio appearances (46%).

In terms of administrative, research, and teaching activities, the majority of the physicians attended health-related administrative meetings online more frequently (70%). However, less than half did more research collaborations by using the phone or the internet (41%) and provided more health-related training and lessons to students online (46%).

### 3.5. Tools Used for Telehealth before the COVID-19 Pandemic

**Figure 3:**
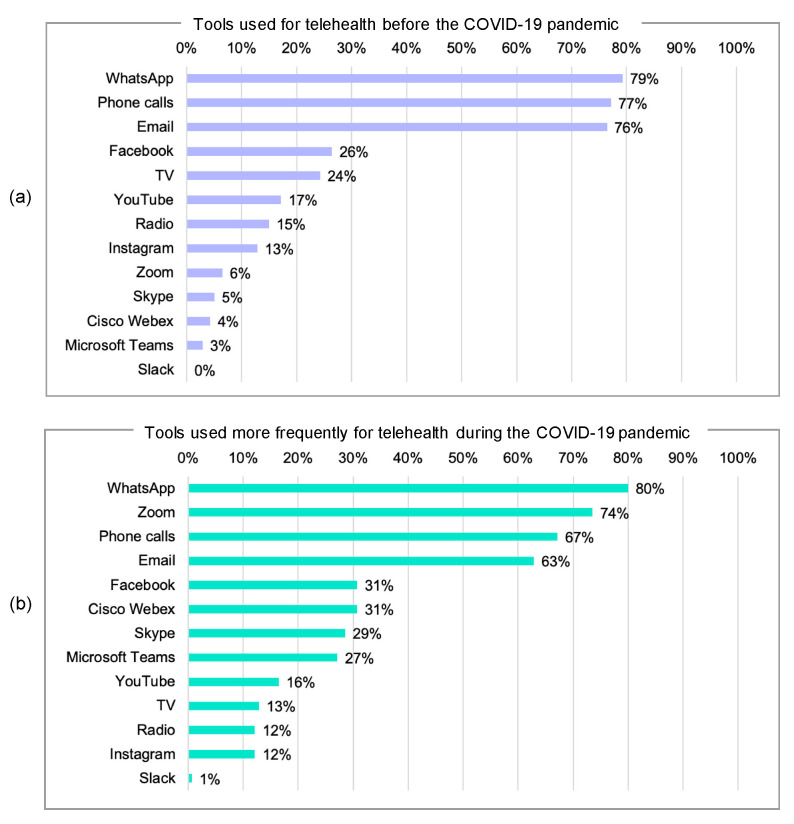
(**a**) Tools used for telehealth before the COVID-19 pandemic; (**b**) tools used more frequently for telehealth during the COVID-19 pandemic.

Figure 3a shows the tools that were used by physicians for telehealth before the COVID-19 pandemic. The numbers indicate the proportion of physicians who used each tool.

Prior to the COVID-19 pandemic, the majority of physicians used WhatsApp (79%), phone calls (77%), and email (76%) for their telehealth activities. Facebook was the most-used social media platform (26%), in comparison with YouTube (17%) and Instagram (13%). A minority used broadcast media platforms, with TV being used more than radio (24% vs. 15%). Very few physicians used video conferencing platforms. Zero physicians reported using Slack prior to the COVID-19 pandemic.

### 3.6. Tools That Were More Frequently Used for Telehealth during the COVID-19 Pandemic

Figure 3b shows the tools that were more frequently used by physicians for telehealth during the COVID-19 pandemic. The numbers indicate the proportion of physicians who increased their use of those tools.

During the COVID-19 pandemic, the majority of physicians increased their use of WhatsApp (80%), phone calls (67%), and email (63%) for their telehealth activities. Moreover, some reported that they increased their use of social media platforms like Facebook (31%), YouTube (16%), and Instagram (12%). The results also show a major increase in the use of video conferencing platforms, with Zoom having the largest increase in use (74%), in comparison with Cisco WebEx (31%), Skype (29%), and Microsoft Teams (27%). A minority increased their telehealth activities on broadcast media platforms (13% on TV, and 12% on radio). Finally, very few physicians reported using Slack during the COVID-19 pandemic (1%).

### 3.7. Perceptions Regarding Telehealth

**Figure 4:**
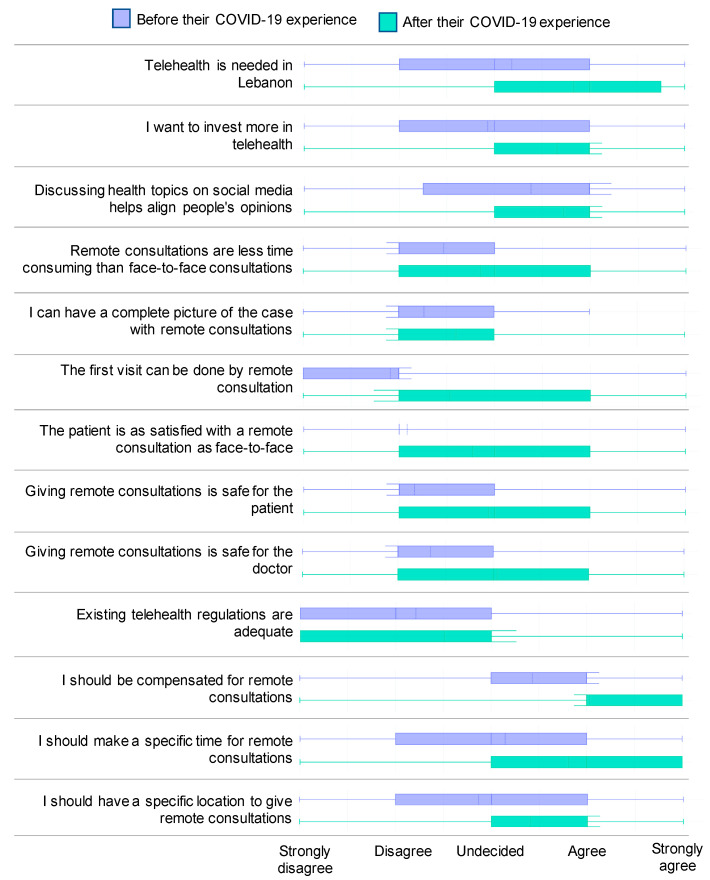
The effect of the COVID-19 pandemic on the physicians’ perceptions regarding telehealth.

Table 3 and Figure 4 show the perceptions of physicians regarding telehealth before and after their experience of providing healthcare during the COVID-19 pandemic. 

The results show that there was a general shift in the physicians’ perceptions regarding telehealth during the COVID-19 pandemic. 

Post-pandemic, physicians agree more on the need for telehealth in Lebanon (U = 6671, *p* < 0.00001) and express a greater willingness to invest in telehealth (U = 6352, *p* < 0.00001). Physicians also agree more that discussing health topics on social media helps align people’s opinions (U = 8320, *p* < 0.00001). 

Regarding the efficiency and effectiveness of remote consultations, there was a significant change in perceptions: Post-pandemic, physicians agree more that remote consultations can be less time-consuming (U = 8360, *p* = 0.00782), that they can get a complete picture of the case (U = 8567, *p* = 0.01778), and that first visits can be done remotely (U = 7107, *p* < 0.00001). Although the perceptions shifted, the physicians who think that remote consultations are less time-consuming than face-to-face consultations (34%), that remote consultations allow them to have the complete picture of the patient’s case (23%), and that the first visit can be done remotely (29%) are still a minority. 

Regarding the safety of and the patient satisfaction with remote consultations, our analysis showed that there was a significant change in perceptions, whereby physicians tended to agree more that remote consultations can be safe for the patients (U = 6323, *p* < 0.00001) and the doctors (U = 6805, *p* < 0.00001), and that patients can be as satisfied with remote consultations as with face-to-face ones (U = 6345, *p* < 0.00001).

Although the experience of providing telemedicine during the COVID-19 pandemic made more physicians feel that telemedicine is safe for the patient (6% before vs. 37% after) and provides equal satisfaction as a face-to-face consultation (5% before vs. 29% after), the majority of physicians still disagrees or remains undecided. On the other hand, a significant number of physicians (41%) started to think that providing remote consultations is safe for the doctor.

We also found a significant change in physicians’ perceptions regarding the management of their telemedicine practice. After their experience during the COVID-19 pandemic, physicians tended to agree more that they should be compensated for their remote consultations (U = 7143, *p* < 0.00001) and that they should have a specific time (U = 6928, *p* < 0.00001) and place (U = 7452, *p* = 0.00008) designated to provide them.

The perception regarding the adequacy of existing telehealth regulations was the only aspect for which no significant shift occurred (U = 8882, *p* = 0.05486). The physicians who think that the existing regulations regarding telehealth are adequate remain a minority (24%).

## 4. Discussion

This work examined the physicians’ use and perceptions of telehealth before and during the COVID-19 pandemic in Lebanon. The findings showed that the COVID-19 pandemic affected the way physicians use and perceive telehealth. The average physician expanded his or her telehealth activities both in type and frequency. This was accompanied by an expansion of the physicians’ repertoire of IT tools and a shift in their perceptions regarding various aspects of telehealth. The experience of providing healthcare during the COVID-19 pandemic seems to have contributed to greater openness and willingness to adopt telehealth in Lebanon. 

In the next sections, we discuss our principal results and their implications for medical practice and policy-making. Finally, we discuss the limitations of our study and propose future research directions. 

### 4.1. Principal Results

Although the use of telehealth was thought to be limited in Lebanon, our study showed that, prior to the COVID-19 pandemic, a large proportion of Lebanese physicians engaged in an array of telehealth activities, such as providing telemedicine services, raising public awareness, and engaging in continuing medical education. Due to the COVID-19 pandemic, Lebanese physicians increased the frequency of their telehealth activities and expanded their scope to include online medical teaching and administrative work.

Prior to the COVID-19 pandemic, Lebanese physicians were engaging in telemedicine, even though they were skeptical of its safety and effectiveness and did not believe that existing telehealth regulations were adequate. Following the experience of providing remote care during the COVID-19 pandemic, a large number of physicians started to believe that telehealth is needed in Lebanon and that they should invest more in telehealth and organize a remote medical practice. This transformation was accompanied by a shift in perception, whereby physicians became more convinced of the efficacy, efficiency, and safety of telemedicine, as well as their ability to satisfy their patients’ needs remotely. These results imply that Lebanese physicians are more open and willing to adopt telemedicine services and would not necessarily return to baseline once the crisis is over. 

Even though the shift in the physicians’ activities and perceptions indicate greater openness and willingness to adopt telehealth services, a significant amount of skepticism and uncertainty regarding telemedicine remains. The majority of physicians disagree, or remains undecided, as to whether remote consultations are as time efficient and effective as face-to-face consultations. Previous studies noted that some physicians worry that telemedicine could undermine their professional autonomy and add to their workload [20]. With face-to-face consultations, the physicians enjoy a high degree of professional autonomy and control over the situation. Indeed, our interviews revealed some of the concerns that physicians have in relation to telemedicine, such as the lack of organization, completeness, remuneration, and flexibility. In terms of organization, one physician was concerned that patients would take advantage of their availability. This can occur when patients have their doctor’s personal contact information, which is common in Lebanon. In terms of completeness, the physicians mentioned that it was impossible to gather all of the needed information during remote consultations, as they could not conduct a thorough clinical examination remotely. Moreover, they are unable to pick up on non-verbal cues, act quickly in case of an emergency, or make sure that the patient fully understands the medical recommendation. In terms of remuneration, the physicians we interviewed were usually providing telemedicine for free, as they viewed it as an extra service that complements their face-to-face consultations. Instead of expecting direct remuneration for their telemedicine services, the physicians may have been using it as a tool to improve their relationships with their patients, as well as improve their professional status [21,22]. Regarding flexibility, one physician mentioned the inflexibility of telemedicine platforms in terms of managing consultation times, which results in dead time. With face-to-face consultations, a consultation that ends early can be followed immediately with a new consultation, as patients are in the waiting room. Moreover, physicians have the option to control the consultation length, depending on their daily workload. However, with telemedicine platforms currently used in Lebanon, physicians can only serve patients during their pre-allocated time slots.

In terms of raising public awareness, 31% of physicians reported increasing their use of Facebook for telehealth purposes. This increase during the COVID-19 pandemic could be attributed to their attempts at establishing a dialogue with the public to communicate the risks, highlight the facts, and provide actionable recommendations to prevent a health disaster in the country [23]. 

In other respects, our results confirmed that the use of video conferencing for telehealth purposes was low prior to the COVID-19 pandemic and dramatically increased during the crisis. This can be attributed to the increased need to provide online medical training, attend medical conferences remotely, and take part in online administrative meetings. It is important to note that the COVID-19 pandemic was preceded by an economic crisis that made it harder for physicians to pay travel costs and find sponsors in order to attend conferences. Therefore, the increase of telehealth activities in terms of continuing medical education could have been amplified by the sudden decrease in financial resources that predated the COVID-19 pandemic. 

Our results also showed that use of all video conferencing platforms increased, but Zoom was by far the most used, even though its security vulnerabilities were widely known [24]. This can be attributed to it being especially user-friendly for first-time users, its newfound visibility nationally and internationally, and its features that could accommodate the majority of the physicians’ telehealth activities.

Regarding regulations, our study did not show a significant change in perceptions due to the crisis. The majority of physicians remains undecided or thinks that existing regulations are inadequate. Still, physicians agreed more that offering telemedicine services is safe for both patients and doctors. This could be explained by the fact that, during the COVID-19 crisis, the Lebanese Order of Physicians and the Ministry of Public Health asked physicians to offer telemedicine services, which may have induced a feeling of safety, even with the lack of adequate regulations. The sudden requirement for telemedicine put the physicians in an unregulated situation; some regulation could have helped them with this transition. Ensuring the continuity of telemedicine activities after the COVID-19 crisis requires adequate regulations, especially considering that the absence of medicolegal frameworks protecting physicians is a major barrier for the adoption for telemedicine [25,26].

Finally, a significant proportion of Lebanese physicians study in Western European and North American countries [27], where their peers engage in remunerated and regulated telemedicine. Moreover, with a long economic crisis on Lebanon’s horizon [28], Lebanese physicians are aspiring to expand their services to nearby Arab countries. Our results show that Lebanese physicians want to be compensated for their remote care, and thus might be more willing to provide telemedicine services if extrinsic rewards are expected [17]. Being aware of the feasibility of telemedicine, the potential of monetizing it, and with a future perspective in mind, Lebanese physicians may have adopted a more accepting attitude toward telemedicine and aim to regulate it.

### 4.2. Implications

Physicians in Lebanon are engaging in telehealth activities without the existence of comprehensive platforms and legal frameworks. Their use of telehealth increased during the COVID-19 pandemic and rendered them more open and willing to engage in telehealth. We offer some practical suggestions that could tackle the barriers and capitalize on the facilitators for telehealth in Lebanon.

From the regulatory perspective, the Lebanese Order of Physicians, the Ministry of Public Health, and the Medical Associations should establish comprehensive medicolegal frameworks to govern telehealth activities. Having these frameworks would protect the patients and the physicians and provide clear guidelines for physicians to adhere to. This is necessary, as the lack of such frameworks is an important barrier for the adoption of telehealth in general and telemedicine in particular. Moreover, it would alleviate the concerns of Lebanese physicians who are witnessing a rise in malpractice lawsuits and attacks by the media that are affecting the already “flawed” doctor–patient relationship [29,30].

In a similar vein, the success of telehealth initiatives requires stakeholders with high levels of e-health readiness, i.e., ones who are capable of successfully adopting and using e-health services. Our study shows that, in general, physicians are open and willing to adopt telehealth. Although these are necessary factors, they are not sufficient to ensure the successful use of telehealth services [31,32,33]. The readiness of other stakeholders—such as patients, their caregivers, and healthcare institutions—also needs to be considered. To assess the stakeholders’ readiness and improve it, localized e-health readiness assessment tools are needed. 

In other respects, as physicians aim to expand their telehealth services inside and outside the country, and the government aims to increase medical tourism, a reliable health IT infrastructure is necessary. Currently, there is a lack of safe, usable, and flexible platforms in the country. Moreover, there are no easy ways to connect Lebanese physicians with patients outside the country. Local IT developers and media can fill in this gap by creating platforms that allow Lebanese physicians to offer remote medical services, provide health awareness and education, engage in international research, and expand the reach of the Lebanese medical sector. To gain an initial understanding of the physicians’ needs, developers can examine how the physicians appropriated already-existing tools [34,35] to support their telehealth activities during the COVID-19 crisis. 

Evidently, faster and cheaper internet plans, in addition to reliable remote payment services, are needed to enable telehealth activities. These two factors may prove to be important barriers in the near future, due to the uncertainty of Lebanon’s economy.

### 4.3. Limitations and Future Research

First, the demographic characteristics of our respondents show a higher proportion of male physicians (66.4% male vs. 33.6% female). A study examining the characteristics of healthcare providers in Lebanon in 1998 showed that fewer than 20% of physicians in Lebanon are female [27]. Since then, the percentage of female physicians has slowly increased, to reach the current estimate of 25%. The slight overrepresentation of female physicians in our sample could be attributed to our sampling method, as many of our questionnaires were sent to physicians in already-existing WhatsApp groups where female and male physicians were almost equally represented. On the other hand, the male doctors were generally more active in the groups, which had become platforms to discuss not only medical information but also to share jokes and political opinions. This might partly explain why more male doctors answered our questionnaire. 

Second, our study was not designed to allow for a subanalysis of factors that may affect the use and perceptions of telehealth, such as the physicians’ medical specialty, age, gender, cultural background, and prior experience. Indeed, the physicians that we interviewed noted that their perceptions of telemedicine in terms of effectiveness, limitations, safety, and satisfaction are highly dependent on their specialty. This aligns with previous studies that showed that different specialties have different levels of satisfaction with telemedicine [36]. Further research is needed to understand how different physicians’ characteristics affect their use of telehealth and their perception of it.

Finally, our study was conducted during the COVID-19 crisis and did not examine whether the change in use and perception of telehealth would outlast the pandemic. Since providing telehealth services was essential to their mission as physicians during the pandemic, the physicians’ willingness to engage in telehealth increased [25]. Once the crisis is over, a reduction in telehealth is expected. Further research is needed to examine the lasting impact of the COVID-19 pandemic on the use and perception of telehealth.

## 5. Conclusions

Our study revealed that physicians in Lebanon increased their telehealth activities during the COVID-19 pandemic in the fields of telemedicine, public awareness, continuing medical education, research, administration, and teaching. This was accompanied by an expansion of their repertoire of information-technology tools and a significant shift in their perceptions regarding various aspects of telehealth. Although the shift in the physicians’ activities and perceptions indicates greater openness and willingness to adopt telehealth services, a significant amount of skepticism and uncertainty regarding telemedicine remains. The two main issues were the effectiveness of remote consultations and the lack of adequate medicolegal frameworks. Based on our findings, we offered recommendations for health IT policy makers, developers, and researchers to sustain the continuity of telehealth activities beyond the COVID-19 pandemic and enable the wide adoption and use of telehealth services.

## Figures and Tables

**Table 1:** The list of themes, subthemes, and supporting quotes from the interviews.

Themes	Subthemes	Quotes from the Interviews
**Telehealth Activities**		
**Telemedicine**	Physicians receive test results from patient over the phone or internet	“Patients sometimes send me their test results when they receive them as a photo over WhatsApp”
	Physicians receive test results from centers over the internet	“I work with a center, and they gave me an ID and password to log in and see the test results”
	Patients consult with physicians over the phone or internet	“I give consultations to Lebanese patients living abroad. They come to the clinic when they are in Lebanon and we continue the follow-up through the phone when they leave Lebanon” “Sometimes the patient’s family sends me videos of the patient having a seizure, which is helpful”
	Physicians prescribe medications to patients over the phone or internet	“I send patient lots of prescriptions through WhatsApp and through email”
**Public Awareness**	Physicians provide public awareness through internet or media channels	“I participate in awareness campaigns on the radio, on TV, and on social media”
**Continuing Medical Education**	Physicians attend health-related webinars/conferences online	“Webinars are time and money sparing tools, we used to travel to attend, now we can easily access them, especially with the high cost associated with conferences”
	Physicians are part of an online group that discusses health-related issues	“I am in many WhatsApp groups with colleagues”
**Research**	Physicians collaborate on health-related research, using the phone or the internet.	“I have been doing a PhD remotely. We have our meetings online and share the data between Lebanon and France”
**Administration**	Physicians attend health-related meetings online	“I participate in a board meeting, with international researchers and practitioners, on a video platform, once a month”
**Teaching**	Physicians give health-related training/lessons to students online	“Before, I didn’t do online teaching. But now we’re obliged to do it”
**Telehealth perceptions**		
**Need for Telehealth**	Telehealth is needed in Lebanon	“I do a little more diagnosis online especially for people from remote areas because they cannot drive to Beirut”
	Wanting to invest in telehealth	“I need to invest more in telehealth, not because of COVID-19. I already had plans to do that anyway before the epidemic” “Interested in using telehealth for international telemedicine and more international research”
**Health-Related Discussions on Social Media**	Discussion of health topics on social media polarizes people’s opinions	“Things get polarized on WhatsApp groups because some people are louder, they chat a lot, send a lot of texts, even when they are wrong, because other people are busy, you hear a lot of echo”
**Effectiveness of Telemedicine**	Inability to get the complete picture of the case with remote consultations	“Triage is more effective face-to-face: once a patient came to my clinic, the moment he walked into the clinic, the color of his face was reddish, he told me he took many pills of aspirin before coming, I sent him directly to the emergency department. Maybe I would not have noticed this with telemedicine. The appearance of the person, his general presentation, is very important”
	The first visit cannot be done remotely	“I don’t usually use telemedicine for the first visit, or to ask for a medical inquiry”
**Efficiency of Telemedicine**	Remote consultations are more time-consuming than face-to-face consultations	“It’s time-consuming”, and “it’s an interruption of daily activities” “We have connection cuts. We stop the call, call again. This is not practical; it takes more time than it should” “The platform that I use is very inflexible. I cannot start a consultation earlier even if the previous one ended prematurely; this creates a lot of dead time”
**Patient Satisfaction with Telemedicine**	Patient satisfaction is different with remote consultation and face to face consultations	“Some patients might think it’s more beneficial, because they have a free medical act over the phone. Some patients might abuse it” “Patient satisfaction is not the same: They do not feel the same, they do not feel like it’s the same kind of service, [and] they are reluctant to pay the same amount for a visit. I think the visit would be shorter. It will be less personal and friendly” “We should ask the patients if they prefer face-to-face consultations, but I think they prefer it, definitely”
**Safety of Telemedicine**	Remote consultations are not always safe for the patients	“Whenever there is any risk in providing telemedicine, I avoid it and tell them I have to see them to examine them or send them to the emergency room” “I have a major concern with children—you have to think about physical abuse with a child, how can I really address it, how can I have a good examination...” “I cannot be sure that the patient fully understands the medical recommendation on the phone. Usually there is a family member with them at the clinic, so I also explain it to them”
	Remote consultations are not always safe for the doctors	“I feel some risk with telemedicine. Before COVID-19 the risk was higher because you were more accountable, but now you will not be held accountable. I think I took a little bit higher risk during COVID-19”
**Medicolegal Aspects**	Existing telehealth regulations are not adequate	“We had to act in an emergency setting despite knowing that there are regulations that are missing” “We should have more regulation, now more than ever, concerning telehealth in general and telemedicine specifically”
**Remuneration for Telemedicine Services**	Remote consultations are offered as an extra free service	“When someone calls for a few minutes, I don’t ask any payment for it” “Someone proposed to pay for the phone consultation, and I refused” “I don’t expect to get paid from patients that I have been seeing for a long time, with the understanding that they would come back and resume their treatment”
**Management of Telemedicine Practice**	Remote consultations are provided at anytime	“If this continues, we would need to do something different, allocate time in the clinic with appointments, and give them a slot on an online platform, and be in the clinic to have the chart in front of us and update the chart”
	Remote consultations are provided anywhere	“I answer the patients whenever I can, from the car, from my home…” “Being at home is not very practical for all kinds of communication”

**Table 2:** Summary of respondent characteristics.

Characteristics		Frequency	Percentage
Age	25–30	2	1.43%
	31–35	12	8.57%
	36–40	44	31.43%
	41–45	40	28.57%
	46–50	10	7.14%
	51–56	10	7.14%
	56–60	11	7.86%
	60–65	9	6.43%
	66+	2	1.43%
Gender	Male	93	66.4%
	Female	47	33.6%
	Other	0	0%
Specialty	Anesthesia	10	7.14%
	Cardiology	3	2.14%
	Dermatology	5	3.57%
	Endocrinology	5	3.57%
	ENT	4	2.86%
	Family medicine	9	6.43%
	Gastroenterology	10	7.14%
	General medicine	2	1.43%
	General surgery	8	5.71%
	Infectious diseases	1	0.71%
	Internal medicine	2	1.43%
	Nephrology	1	0.71%
	Neurology	3	2.14%
	Obstetrics and Gynecology	11	7.86%
	Oncology	8	5.71%
	Ophthalmology	1	0.71%
	Orthopedics	5	3.57%
	Osteopathic medicine	1	0.71%
	Pediatric surgery	1	0.71%
	Pediatrics	21	15.00%
	Plastic surgery	4	2.86%
	Pneumology	2	1.43%
	Psychiatry	11	7.86%
	Radiology	5	3.57%
	Rheumatology	1	0.71%
	Urology	5	3.57%
	Vascular and thoracic surgery	1	0.71%

**Table 3:** Physicians’ perceptions of telehealth before and after their experience of the COVID-19 pandemic.

Perceptions		Strongly Disagree	Disagree	Undecided	Agree	Strongly Agree	Mann–Whitney U test
Telehealth is needed in Lebanon	Before	7%	21%	30%	31%	11%	*z*-score = −5.1, *p* < 0.00001, U = 6671
	After	6%	5%	16%	49%	25%	
I want to invest more in telehealth	Before	7%	31%	29%	27%	6%	*z*-score = −5.5, *p* < 0.00001, U = 6352
	After	6%	8%	21%	45%	20%	
Discussing health topics on social media helps align people's opinions	Before	5%	20%	16%	51%	9%	*z*-score = −5.1, *p* < 0.00001, U = 8320
	After	4%	11%	14%	48%	22%	
Remote consultations are less time-consuming than face-to-face consultations	Before	12%	50%	21%	14%	4%	*z*-score = −2.7, *p* = 0.00782, U = 8360
	After	8%	41%	16%	27%	7%	
I can have a complete picture of the case with remote consultations	Before	21%	46%	20%	14%	0%	*z*-score = −2.4, *p* = 0.01778, U = 8567
	After	13%	41%	23%	18%	5%	
The first visit can be done by remote consultation	Before	36%	45%	13%	4%	2%	*z*-score = −4.5, *p* < 0.00001, U = 7107
	After	19%	40%	11%	27%	2%	
The patient is as satisfied with a remote consultation as face-to-face	Before	23%	53%	19%	4%	1%	*z*-score = −5.5, *p* < 0.00001, U = 6345
	After	11%	33%	27%	28%	1%	
Remote consultations are safe for the patient	Before	21%	49%	23%	5%	1%	*z*-score = −5.6, *p* < 0.00001, U = 6323
	After	12%	28%	24%	28%	9%	
Remote consultations are safe for the doctor	Before	21%	41%	24%	10%	4%	*z*-score = −4.9, *p* < 0.00001, U = 6805
	After	10%	28%	21%	30%	11%	
Existing telehealth regulations are adequate	Before	29%	36%	24%	11%	1%	*z*-score = −1.9, *p* = 0.05486, U = 8882
	After	29%	20%	27%	19%	5%	
I should be compensated for remote consultations	Before	7%	17%	21%	34%	20%	*z*-score = −4.4, *p* < 0.00001, U = 7143
	After	4%	8%	10%	36%	41%	
I should make a specific time for remote consultations	Before	9%	24%	22%	34%	11%	*z*-score = −4.7, *p* < 0.00001, U = 6928
	After	5%	6%	18%	42%	29%	
I should have a specific location to give remote consultations	Before	11%	27%	31%	28%	4%	*z*-score = −4, *p* = 0.00008, U = 7452
	After	8%	13%	27%	34%	18%	

## Appendix A. Semi-Structured Interview Guide

***Participants***

Lebanese physicians

***Introduction***

Thank you for agreeing to participate in this interview. We are interviewing you to better understand your use and perceptions of telehealth prior to, and during, the COVID-19 pandemic.

The interview should take approximately 20 min. If it is okay with you, I would like to record the interview to make sure that we do not miss any of the information you share with us.

The content of the interview will be kept confidential. This means that we will only share the recording, in an anonymized way, with researchers who are working on this project. In addition, we will ensure that, in our report, the information we share will keep your identity anonymous.

You can refuse to answer any question or stop the interview at any time and for any reason. Do you have any questions before we start?

May I start recording the interview now?

***Establishing common understanding of telehealth***

- Can you please tell me about your understanding of what telehealth is?

“Telehealth is different from telemedicine because it refers to a *broader scope of remote healthcare services* than telemedicine. While telemedicine refers specifically to remote clinical services, telehealth can refer to remote non-clinical services, such as provider training, administrative meetings, and continuing medical education, in addition to clinical services [37]”.

***Understanding how they use telehealth***

- What kind of telehealth have you done before the COVID-19 pandemic?
- What kind of telehealth have you done during the COVID-19 pandemic?
- What tools did you use for telehealth before the COVID-19 pandemic?
- What tools did you use for telehealth during the COVID-19 pandemic?
- What were the problems that you encountered while doing telehealth during the COVID-19 pandemic?

***Understanding how they use telemedicine***

- When and where were you providing telemedicine from before the COVID-19 pandemic?
- When and where were you providing telemedicine from during the COVID-19 pandemic?
- Have you ever been paid for a telemedicine service before the COVID-19 pandemic and how?
- Have you ever been paid for a telemedicine service during the COVID-19 pandemic and how?

***Understanding their perceptions of telehealth***

- What was your opinion regarding telehealth before the COVID-19 pandemic?
- Did your opinion change after the COVID-19 pandemic?
- Are you going to invest more of your time in telehealth once the crisis is over? Or will you return to baseline?
- What kind of telehealth are you thinking of investing in in the future?

***Understanding their perceptions of telemedicine***

- Did you think telemedicine is equally effective as a face-to-face consultation before the COVID-19 pandemic?
- Did your opinion change after the COVID-19 pandemic?
- Did you think the patients’ satisfaction is the same with telemedicine as with a face-to-face consultation?
- Did your opinion change after the COVID-19 pandemic?
- What are the situations in which you wouldn't accept to use telemedicine?
- Were these situations different during the COVID-19 pandemic?
- How much would you price a telemedicine consultation in comparison to a face to face consultation and why?
- Are you aware of any law in relation to telemedicine?
- Does the law still apply during the COVID-19 pandemic?
- Do you think we need stricter or less strict laws around telemedicine?
- Did you feel you are taking a higher risk when providing telemedicine before the COVID-19 pandemic?
- Did you feel you are taking a higher risk when providing telemedicine during the COVID-19 pandemic?

***Conclusion***

Is there anything else that you would like to add or comment on?

Thank you very much for your time and participation.
